# Integrative analysis reveals the potential prognostic roles and immunological values of unc-5 netrin receptor A (UNC5A) in glioma

**DOI:** 10.1007/s12672-024-01174-y

**Published:** 2024-07-22

**Authors:** Wenbo Qian, Lei Zhang, Fenglin Zhang, Jingliang Ye, Zhiping Wan, Huairui Chen, Chun Luo

**Affiliations:** Department of Neurosurgery, Tongji Hospital of Tongji University, School of Medicine, Tongji University, Putuo District, No. 389 Xincun Road, Shanghai, 200092 China

**Keywords:** Glioma, Biomarker, Prognosis, UNC5A, Immunity

## Abstract

**Background:**

UNC5A had been reported to play crucial roles in multiple cancers. However, little was known about the associations among UNC5A and glioma. Therefore, we first combined scRNA-seq, proteomics, as well as bulk RNA-seq in order to investigate UNC5A’s functions in gliomas.

**Methods:**

Online databases provided scRNA-seq, proteomics, as well as bulk RNA-seq data on UNC5A in gliomas. The following procedures were conducted in order: QRT-PCR, Norman chart, gene set enrichment analysis (GSEA), and univariate/multifactor Cox regression analyses. We further explored the associations among UNC5A and tumor immunity.

**Results:**

By comparing gliomas with normal tissues, the TCGA dataset showed a significantly reduced expression of UNC5A, which was also confirmed by GSE50161, GSE4290, and QRT-PCR findings (p < 0.05). In both the TCGA and CGGA datasets, gliomas patients with low-UNC5A expression would have poorer overall survival (OS) prognoses (p < 0.05). ScRNA-seq analysis by the CancerSEA online website presented that UNC5A had a low expression in various glioma clusters and significantly associated with six functional states. Moreover, UNC5A might be a reliable independent biomarker of OS in gliomas patients (p < 0.05). Based on the results of GSEA, UNC5A might be connected to three significant pathways in gliomas. We also successfully created a Norman chart to assess the OS prognoses of these patients. Additionally, in aspects of tumor immunity, the infiltration levels of immune cells in LGG, the immune cell pathways, tumor immune microenvironment, as well as immune checkpoints in both LGG and GBM were revealed to be significantly influenced by UNC5A (p < 0.05).

**Conclusions:**

UNC5A was found to have prognostic and immunological significance in gliomas, offering patients with gliomas new treatment options.

## Introduction

As a primary brain tumor that is highly recurrent or lethal, gliomas are most likely to develop in adults. They are also prone to resistance to apoptosis, genomic instability, invasion, and migration, all of which contribute to a poor prognosis for the disease and have a detrimental emotional and daily impact on patients [1]. The World Health Organization categorized gliomas into grades I–IV, with glioblastoma (GBM) being the most frequent and severe kind, and low-grade glioma (LGG), a heterogeneous collection of tumors that make up about 20% of all primary brain tumors. Despite the fact that LGGs are often dormant, a significant number of them have the potential to develop into deadly high-grade gliomas like GBMs [2]. Furthermore, glioma therapies are more challenging due to the fact that glioma stem cells (GSCs) are a subset of glioma cells that have the capacity to self-renew, resulting in robust resistance to radiation and chemotherapy [3]. As a result, it has become an inevitable trend to study the underlying pathogenesis of glioma to better advance clinical drugs’ development.

Unc-5 netrin receptor A (UNC5A), among the family of netrin-1 receptors, was reported to be able to mediate the chemical pulse effect of netrin-1 on specific axons [4, 5]. UNC5A’s function was a two-edged sword that depended on the availability of ligands. In the absence of ligands, UNC5A was in monomeric form, starting the downstream cascade that induced apoptosis. UNC5A forms dimers in the presence of ligands that convey diverse signals for cell migration, survival, and differentiation [5, 6]. Moreover, it had been shown that UNC5A had a negative feedback regulatory effect in a variety of cancers and the expression of this gene was significantly down-regulated in bladder, rectal and breast cancers, even in liver cirrhosis [7, 8]. In the studies of bladder cancer, a study by Zhu et al. suggested that UNC5A could be a direct target gene for p53, forecasting responses to cisplatin-induced DNA damage and resulting in cell death in this cancer [9]. Thiebault et al. also showed experimentally that the loss of UNC5A might be related to the absence of p53 activity, inducing tumor development [9, 10]. At present, there was still a gap in the researches about the roles of UNC5A in glioma. Therefore, in this study, we first combined single-cell RNA-Sequencing (scRNA-seq), proteomics, and bulk RNA-Sequencing (RNA-seq) in order to investigate UNC5A's expression, prognosis, associated pathways or drugs, and clinical or immune relevance in gliomas, laying the groundwork for UNC5A’s future related studies in gliomas.

## Materials and methods

### Data collecting and processing

From the Chinese Glioma Genome Atlas (CGGA, https://www.cgga.org.cn; glioma = 929), we gathered bulk RNA-seq matrix as well as clinical information of gliomas, including GBM and LGG. Gene Expression Omnibus (GEO), including GSE4290 (Normal = 27; glioma = 153) and GSE50161 (Normal = 13; glioma = 102) datasets, and The Cancer Genome Atlas (TCGA, https://portal.gdc.cancer.gov/, GBM = 163; LGG = 518) datasets were used as external validation datasets. By means of CGGA data mining, UNC5A was finally identified by us in glioma after a series of gene filtrations, including survival analysis, univariate analysis, multivariate analysis, other dataset validations, and polymerase chain reaction (PCR) validations, sequentially. The R 4.1.1 software was applied to handle all the data. UNC5A’s expression and survival differences were determined by the “limma” package, with a threshold of P values < 0.05.

### Quantitative Real Time PCR (QRT-PCR)

Glioma cell lines, including HA-1800 (human normal astrocytes), U87 (human astroblastoma), and U251 (human glioma) cells, were cultured as described in the article [11], and total RNA from these three cell lines was extracted by Trizol (ThermoFisher Scientific) [12, 13]. Then, QRT-PCR was conducted to detect UNC5A’s mRNA expression levels in these three cell lines and analyzed by the 2^−ΔΔCt^ method. The forward and reverse primers of UNC5A from 5′ to 3′ were “AACATTTCTGTGCCTGCTGGGTAG” and “GTGGTGGTCGTGTGCCTGAATC”, respectively. The forward and reverse primers of GAPDH from 5′ to 3′ were “GCCAAGGTCATCCATGACAACTTTGG” and “GCCTGCTTCACCACCTTCTTGATGTC”, respectively.

### CancerSEA, UALCAN, CellMiner online databases

CancerSEA, an online website, was applied to examine UNC5A scRNA-seq data in gliomas (http://biocc.hrbmu.edu.cn/CancerSEA/) to seek its associations with 14 kinds of functional states, such as angiogenesis, cell cycle, DNA damage, EMT, inflammation, quiescence, apoptosis, differentiation, DNA repair, hypoxia, invasion, proliferation, and stemness [14]. The UALCAN online website (http://ualcan.path.uab.edu/index.html) presented the UNC5A proteomics data in GBM to identify the expression differences in gliomas and normal samples, in different genders, ages, weight situations, and in different HIPPO and mTOR pathway statuses through CPTAC analysis [15]. The CellMiner online dataset (http://discover.nci.nih.gov/CellMiner/) was also applied by us to identify UNC5A-significantly related drug sensitivities [16].

### Univariate/multivariate analysis, Norman chart construction and Gene set enrichment analysis

According to the expression of UNC5A and several clinicopathological factors, univariate and multivariate Cox risk analyses were conducted to identify independent biofactors for overall survival (OS) in gliomas, with a threshold of P value below 0.05 [17], and the Norman chart was also constructed to assess the OS prognosis in glioma patients, evaluating it by calibration plots, C-index, and ROC curves using the R “rms” package [18]. Gene set enrichment analysis (GSEA) was also performed to seek UNC5A significantly enriched pathways, with the threshold of nominal P values < 0.05 and |Normalized Enrichment Score (NES)|> 1.5 [19].

### Relationships among UNC5A and tumor immunity, tumor neoantigen load (TNB), tumor mutational load (TMB), microsatellite instability (MSI) in gliomas

As described in previous articles [20, 21], we used Spearman's approach to examine the associations between UNC5A expression and MSI, TMB, and TNB in LGG or GBM via the Sangerbox online platform (http://www.sangerbox.com/tool), with P values < 0.05. Meanwhile, UNC5A’s associations with the tumor immune microenvironment, checkpoints, and immune cell pathways in LGG or GBM were also evaluated by this online tool [22]. The TIMER database (https://cistrome.shinyapps.io/timer/) online website was also applied by us to assess UNC5A’s associations with immune cell infiltration levels in both LGG and GBM [23].

## Results

### UNC5A’s mRNA expression levels and OS prognosis in gliomas

**Fig. 1:**
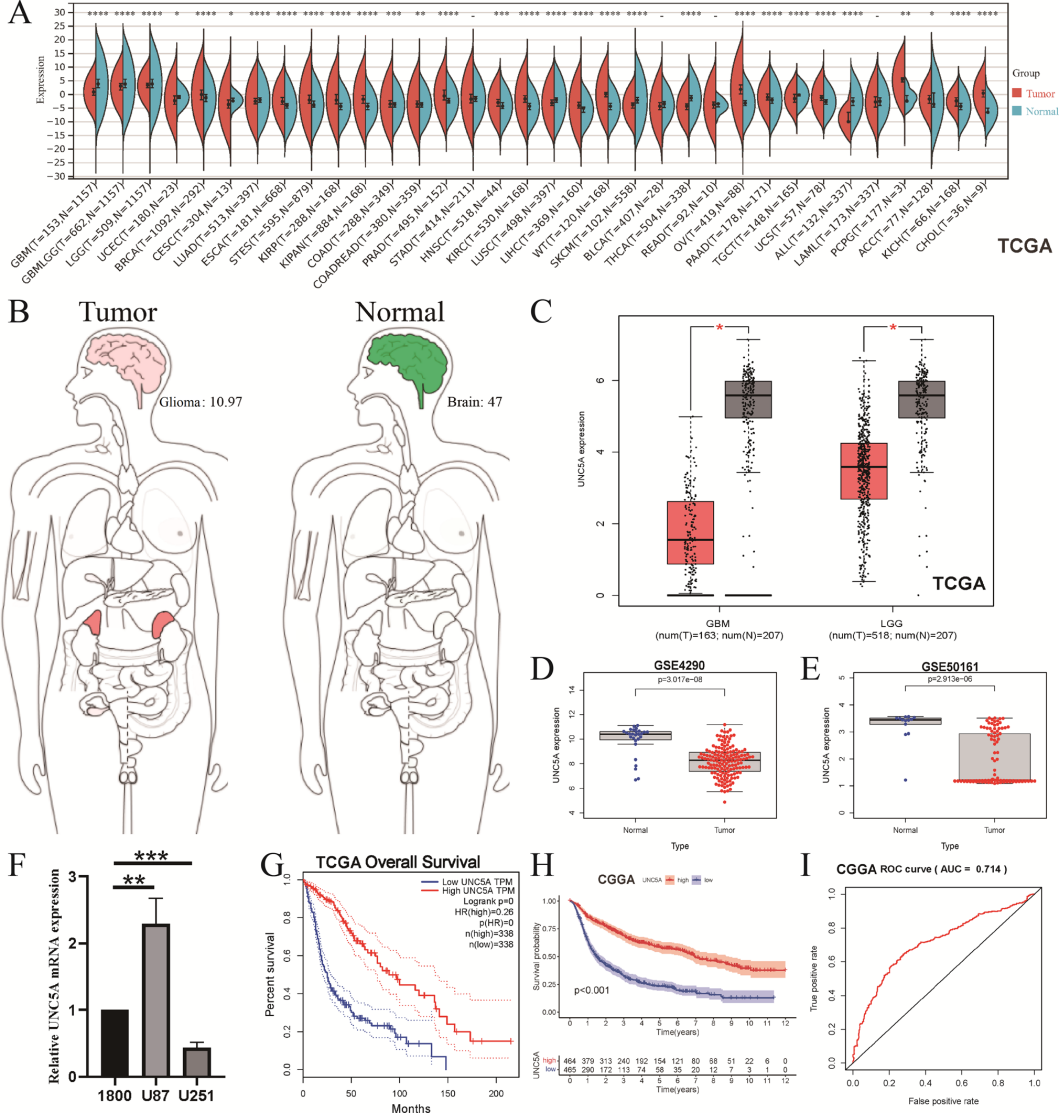
The mRNA expression level of UNC5A in glioma patients correlates with prognosis; **A** Pan-cancer analysis; **B** Human body heatmap; **C** TCGA boxplot; **D** GSE4290 boxplot; **E** GSE50161 boxplot; **F** QRT-PCR results; **G** OS curve in TCGA; **H** OS curve in CGGA; **I** ROC curve in CGGA; *p < 0.05; **p < 0.01; ***p < 0.001; ****p < 0.0001

By TCGA pan-cancer analysis, we observed that UNC5A’s mRNA expression levels were differentially expressed in various cancers, containing glioma samples of LGG and GBM (Fig. 1A). According to the human body heatmap in Fig. 1B, it was evident that UNC5A was less abundant in glioma tissues than in normal samples. In the GSE4290, GSE50161, and TCGA datasets, our results confirmed that UNC5A was lowly expressed in glioma tissues compared with normal tissues, respectively (Fig. 1C–E). Furthermore, UNC5A mRNA levels were substantially reduced in glioma U251 cells, as demonstrated by QRT-PCR data (Fig. 1F). UNC5A’s survival analyses in the TCGA and CGGA datasets both showed that high-UNC5A-expressed groups would have better OS prognoses than those in low-UNC5A-expressed groups (Fig. 1G, H). ROC curves indicated a moderate diagnosis power of UNC5A in gliomas with AUC values of 0.714 (Fig. 1I).

### UNC5A’s scRNA-seq data in gliomas

**Fig. 2:**
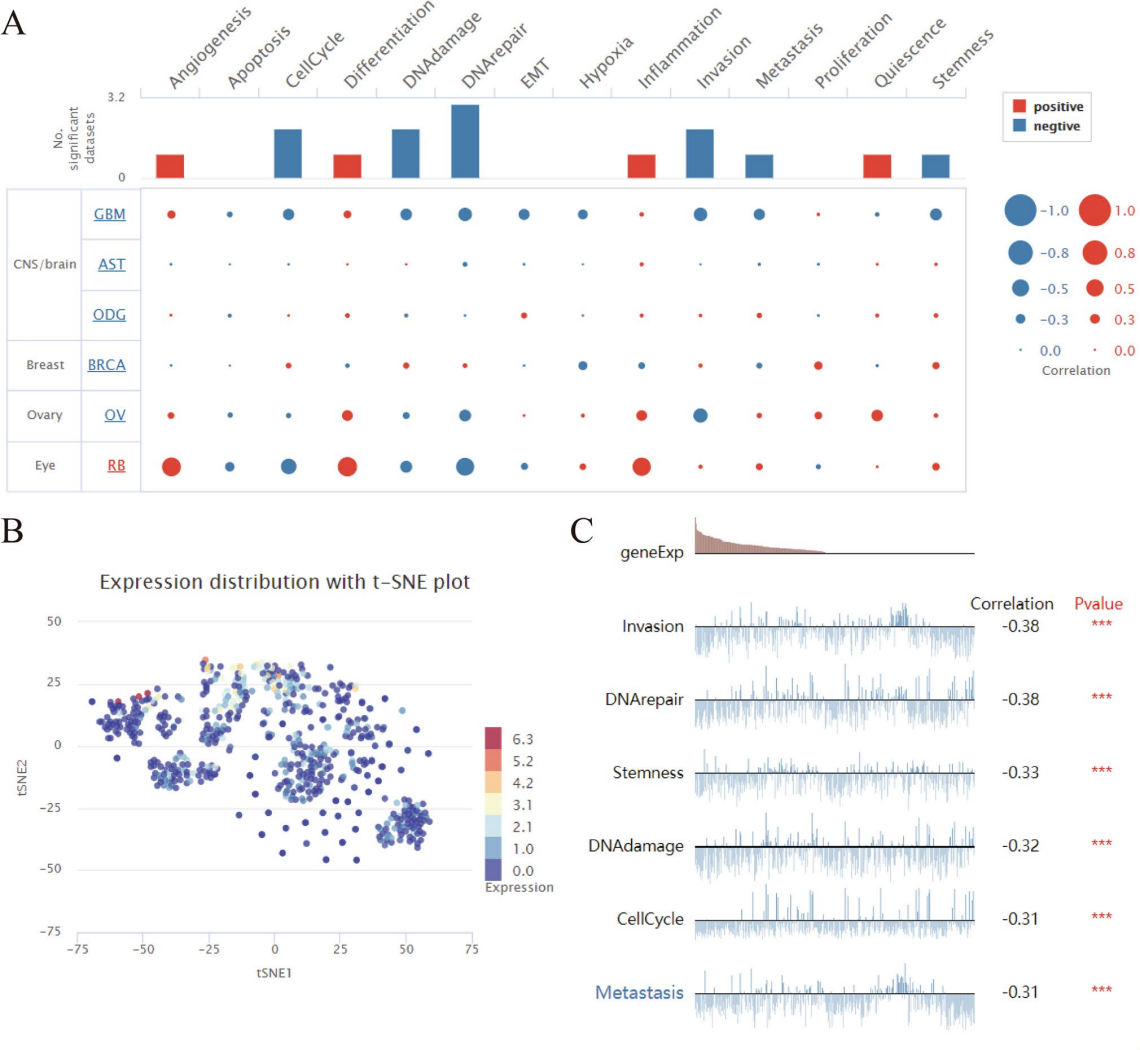
ScRNA-seq data of UNC5A in glioma; **A** Bubble chart of UNC5A’s associations with 14 kinds of functional states; **B** UNC5A’s tSNE plot in glioma clusters; **C** UNC5A significantly associated with six functional states; ***p < 0.001

ScRNA-seq data of UNC5A in gliomas was conducted by the CancerSEA online website to seek its associations with 14 kinds of functional states, such as angiogenesis, cell cycle, DNA damage, EMT, inflammation, quiescence, apoptosis, differentiation, DNA repair, hypoxia, invasion, proliferation, metastasis, and stemness, as displayed by the bubble chart (Fig. 2A). The TSNE plot showed that UNC5A was lowly expressed in various glioma clusters (Fig. 2B). Besides, UNC5A was found to be significantly associated with invasion, DNA repair, stemness, DNA damage, cell cycle, and metastasis functional states (Fig. 2C).

### UNC5A’s protein expression levels in gliomas

**Fig. 3:**
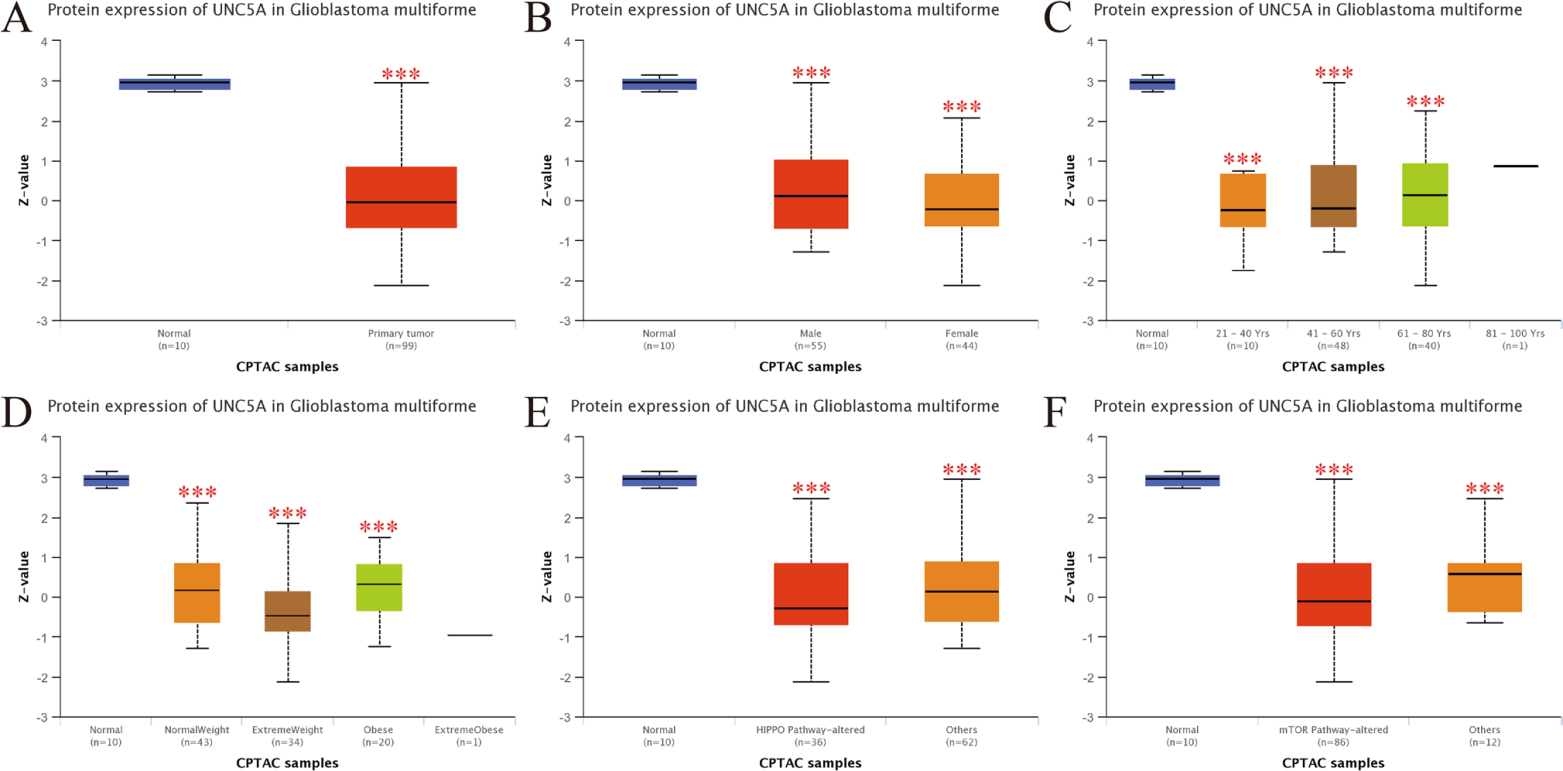
Protein expression of UNC5A in glioma by different subgroups; **A** Tumor or normal type; **B** Gender; **C** Age; **D** Weight; **E** HIPPO pathway; **F** mTOR pathway; ***p < 0.001

The UALCAN online website (http://ualcan.path.uab.edu/index.html) presented the UNC5A proteomics data in GBM to identify the expression differences in gliomas and normal samples (p < 0.001; Fig. 3A), in different genders (p < 0.001; Fig. 3B), ages (p < 0.001; Fig. 3C), weight (p < 0.001; Fig. 3D) situations, and in different HIPPO and mTOR pathway statuses (p < 0.001; Fig. 3E, F) through CPTAC analysis. Consistent with the above-mentioned results, UNC5A’s protein expression levels were also less abundant in gliomas tissues than in normal samples.

### UNC5A’s relationships with clinical features

**Fig. 4:**
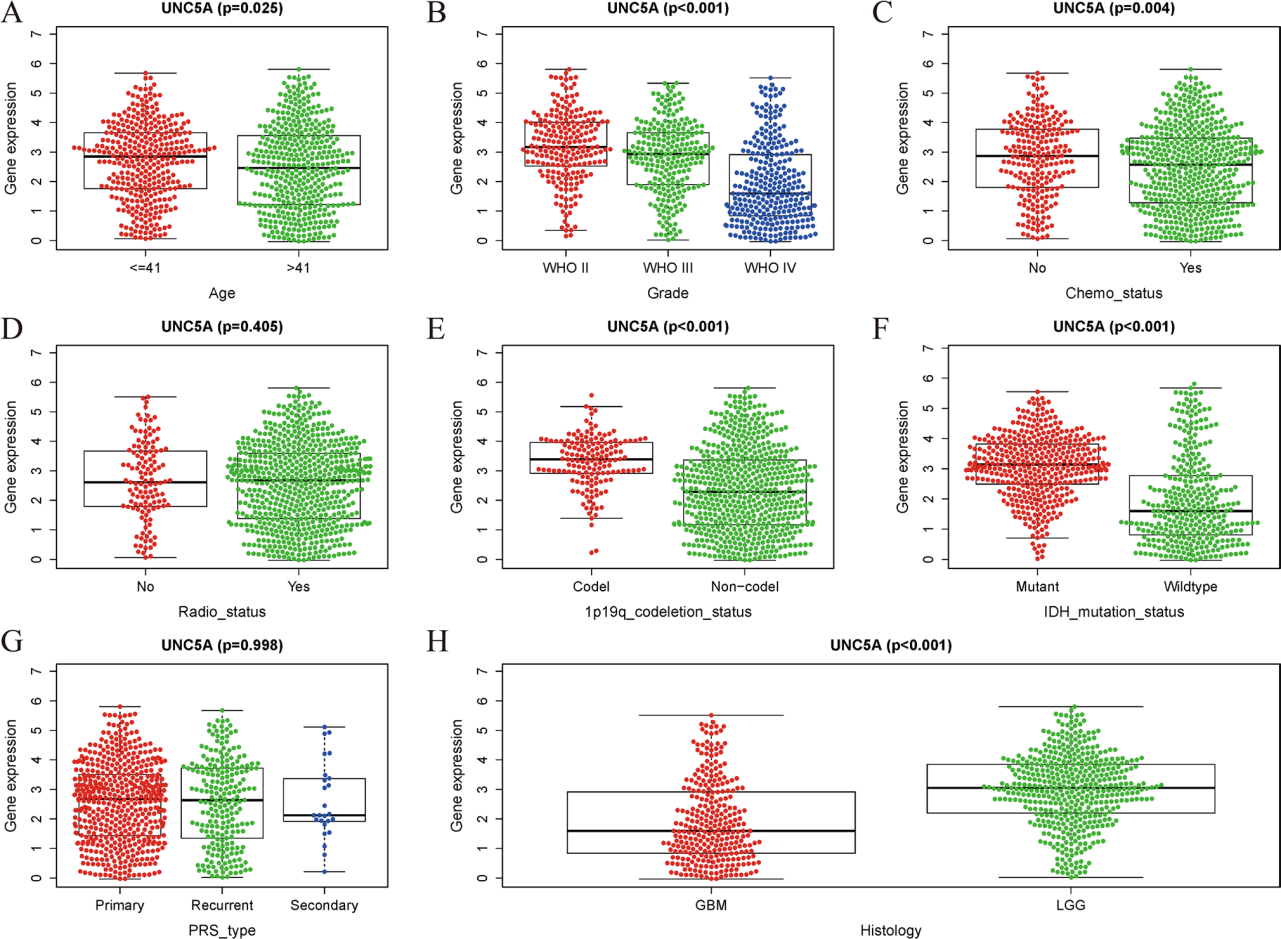
Relationships among UNC5A and clinical features; **A** Age; **B** Grade; **C** Chemo status; **D** Radio status; **E** 1p19q codeletion status; **F** IDH mutation status; **G** PRS type; **H** Histology

Through examination of the RNA-seq matrix as well as clinical information pertaining to gliomas, we discovered that there were statistically significant differences in UNC5A expression with age (p = 0.025), grade (p < 0.001), chemo status (p = 0.004), 1p19q codeletion status (p < 0.001), IDH mutation (p < 0.001), as well as histology (p < 0.001), while it was not significant in gender, radio status (p = 0.405), or PRS type (p = 0.998) (Fig. 4).

### UNC5A’s prognostic roles in gliomas by univariate/multivariate analysis and the Norman chart

**Fig. 5:**
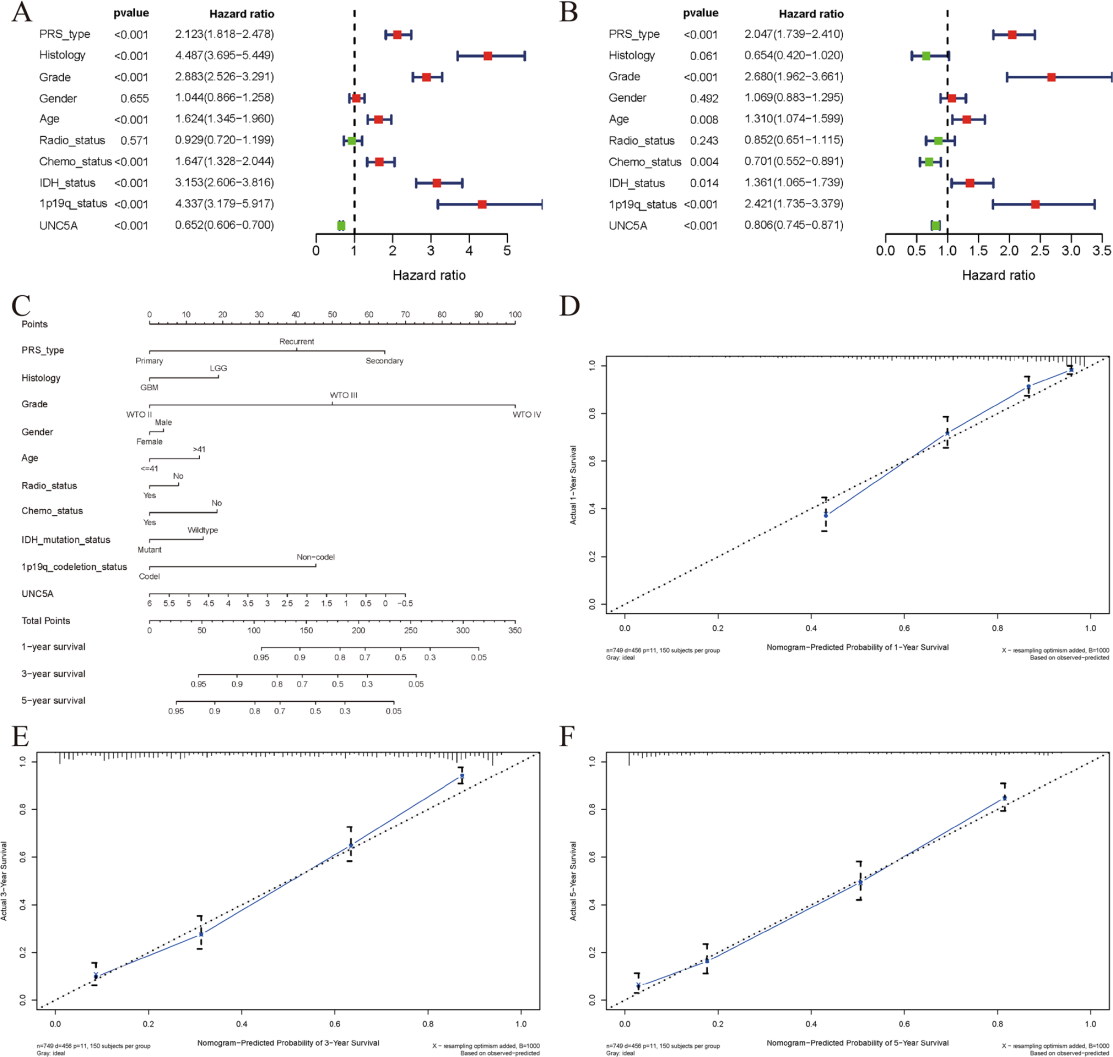
UNC5A could be an independent factor affecting glioma’s prognosis and Norman chart establishment; **A** Univariate Cox regression analysis; **B** Multivariate Cox regression analysis; **C** Norman chart for predicting the overall survival of glioma patients based on clinical parameters and UNC5A expression; **D**–**F** 1-year, 3-year, and 5-year calibration plots

According to univariate analysis, chemotherapy status, age, grade, IDH mutation status, PRS type, 1p19q codeletion status, UNC5A expression, and histology were all significantly associated with OS in gliomas (all p < 0.05; Fig. 5A and Table 1). Based on multivariate analysis, grade, age, IDH mutation status, chemotherapy status, UNC5A expression, 1p19q codeletion status, and PRS type were all shown to be independent predictors of OS in glioma patients (all p < 0.05; Fig. 5B and Table 1). In order to give a quantitative way for predicting the 1-, 3-, and 5-year OS rates in glioma patients, we also created a Norman chart incorporating UNC5A expression and clinicopathological characteristics, evaluating it by calibration plots, C-index, and ROC curves (Fig. 5C). Our established Norman chart’s AUC value of the ROC curve at one year was 0.858, at three years it was 0.880, at five years it was 0.880, as well as its C-index value was 0.800 (Table 2). In addition, calibration plots indicated a good performance of this Norman chart (Fig. 5D–F).

**Table 1:** Univariate and multivariate analyses of UNC5A gene and clinical factors in glima

Clinical factors	Univariate analysis				Multivariate analysis			
	HR	HR.95L	HR.95H	p-value	HR	HR.95L	HR.95H	p-value
PRS_type	2.122582	1.817807	2.478456	1.79E−21	2.047098	1.739015	2.409761	7.37E−18
Histology	4.486991	3.695058	5.448654	7.38E−52	0.654358	0.419987	1.019518	0.060858
Grade	2.883411	2.526415	3.290853	1.44E−55	2.67978	1.961766	3.66059	5.85E−10
Gender	1.04351	0.865536	1.258081	0.655307	1.069351	0.883057	1.294946	0.492363
Age	1.623833	1.345161	1.960236	4.49E−07	1.310271	1.073638	1.599057	0.007834
Radio_status	0.928909	0.719933	1.198546	0.570623	0.851621	0.650524	1.114884	0.242537
Chemo_status	1.647389	1.327807	2.043888	5.71E−06	0.700962	0.551729	0.890561	0.003627
IDH_status	3.153004	2.605537	3.815503	3.84E−32	1.361348	1.065415	1.739479	0.013639
1p19q_status	4.336988	3.179033	5.916724	2.08E−20	2.421013	1.734511	3.379226	2.03E−07
UNC5A	0.6515	0.606086	0.700318	3.17E−31	0.805717	0.7452	0.871149	5.87E−08

**Table 2:** C-index and 1-, 3-, 5-year ROC of the established nomogram

Variables	1_year_ROC	3_year_ROC	5_year_ROC	C-index
Values	0.858	0.880	0.880	0.800

### UNC5A involved pathways in gliomas by GSEA

**Fig. 6:**
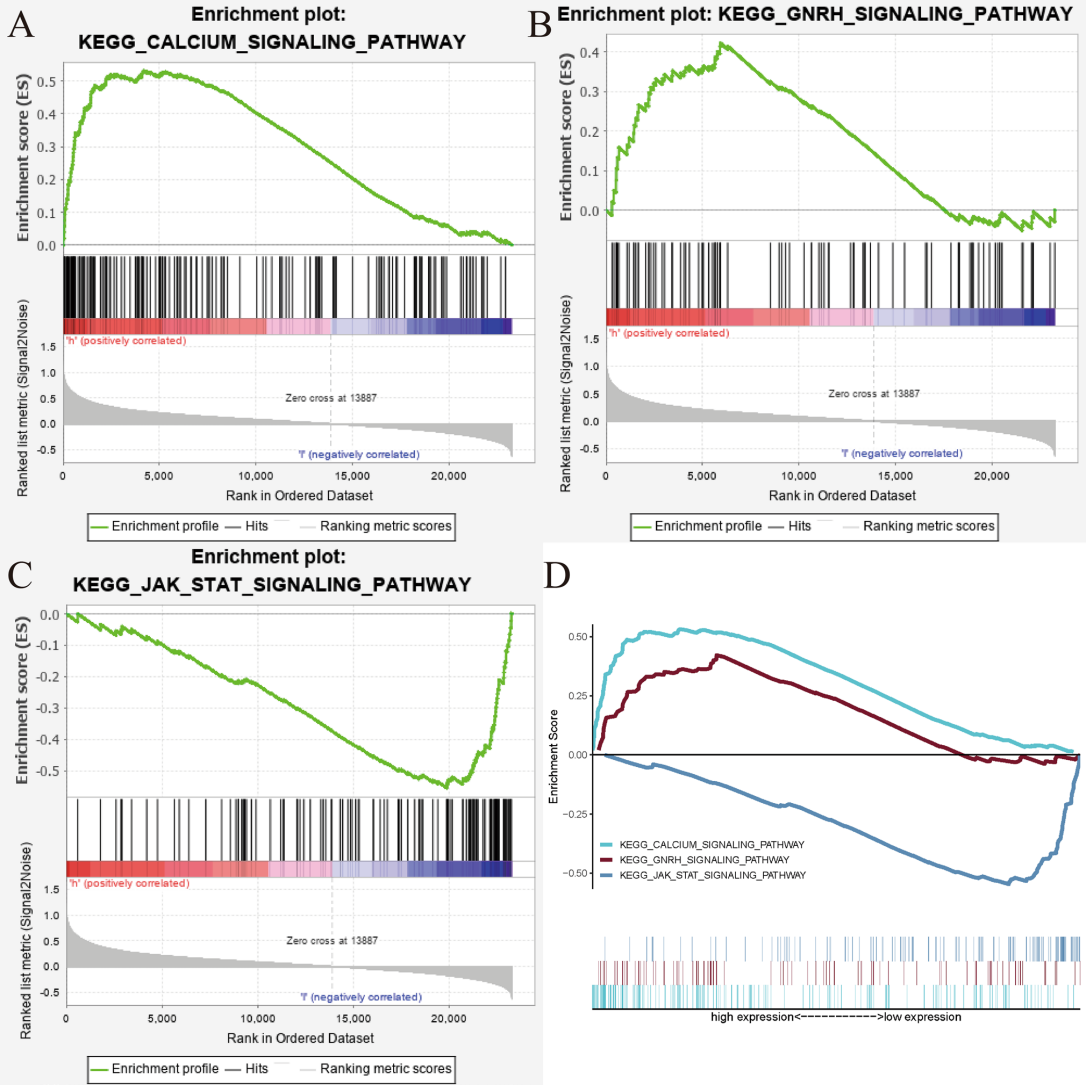
Signaling pathways of UNC5A that might be involved in glioma by gene set enrichment analysis; **A** Calcium signaling pathway; **B** Gnrh signaling pathway; **C** Jak-stat signaling pathway; **D** All of three above mentioned signaling pathways

GSEA was performed to seek UNC5A-significantly enriched pathways among low-UNC5A and high-UNC5A datasets retrieved from the CGGA databases, with the threshold of nominal P values < 0.05 and |NES|> 1.5. Our results revealed that UNC5A might be implicated in three major signaling pathways: the Gnrh signaling pathway, the calcium signaling pathway, as well as the Jak-stat signaling pathway (Fig. 6 and Table 3).

**Table 3:** Gene set enrichment analysis (GSEA) results

GeneSet name	NES	Nominal p-value	FDR q-value
KEGG_calcium_signaling_pathway	1.813	0.006	0.065
KEGG_GNRH_signaling_pathway	1.527	0.042	0.247
KEGG_JAK_STAT_signaling_pathway	− 1.773	0.016	0.04

*NES* normalized enrichment score

### UNC5A’s relationships with the PPI network, TNB, MSI, and TMB

**Fig. 7:**
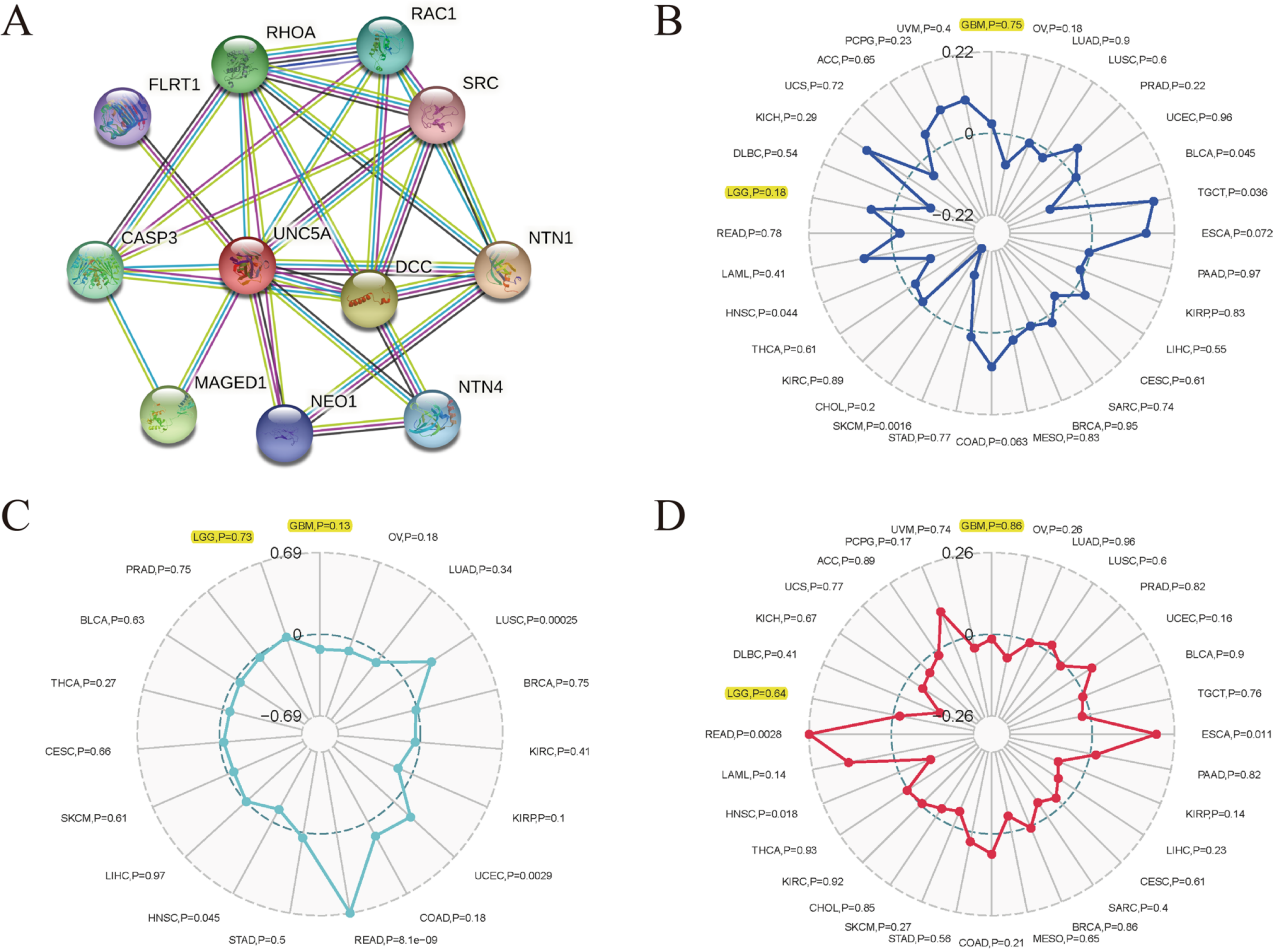
Construction of UNC5A PPI network in glioma and its associations with MSI, TMB, and TNB; **A** PPI networks associated with UNC5A; **B** Relevance of UNC5A to MSI; **C** Relevance of UNC5A to TNB; **D** Relevance of UNC5A to TMB

Through the String (https://string-db.org/) online website, we identified additional proteins, FLRT1, RHOA, RAC1, SRC, CASP3, DCC, NTN1, MAGED1, NEO1, and NTN4, that might be connected to UNC5A and displayed them as PPI networks (Fig. 7A). We used Spearman's approach to examine the associations between UNC5A expression and MSI, TMB, and TNB in LGG or GBM via the Sangerbox online platform (http://www.sangerbox.com/tool), with P values < 0.05. However, no significant correlations were revealed (Fig. 7B–D).

### UNC5A’s immune relevance in gliomas

**Fig. 8:**
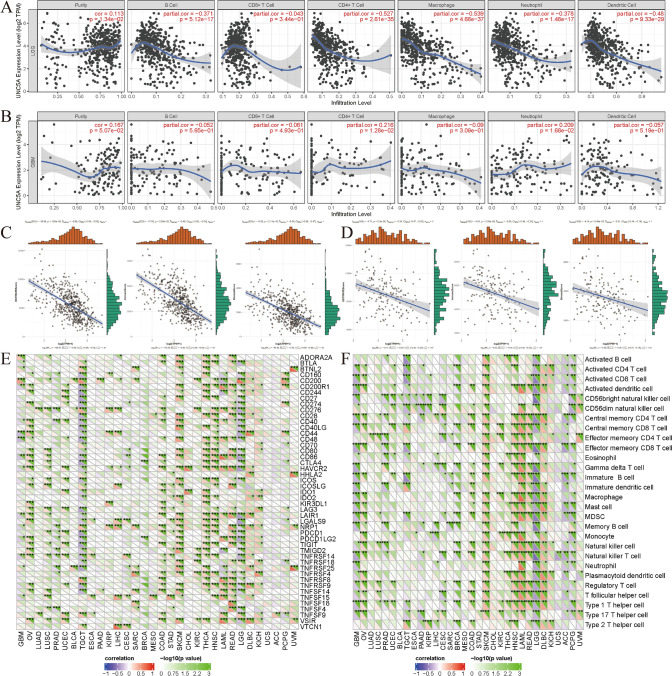
Immunological features of UNC5A in glioma patients; **A** Associations between UNC5A and immune infiltration in LGG; **B** Associations between UNC5A and immune infiltration in GBM; **C** Associations between UNC5A and tumor microenvironment in LGG; **D** Associations between UNC5A and tumor microenvironment in GBM; **E** Expression of UNC5A-related immune checkpoint genes in different tumors; **F** Expression of UNC5A-related immune cells pathways in different tumors. *p < 0.05; **p < 0.01; ***p < 0.001

The TIMER database (https://cistrome.shinyapps.io/timer/) online website was also applied by us to assess UNC5A's associations with immune cell infiltration levels in both LGG and GBM. UNC5A showed significant associations with CD4+T cells, macrophages, B cells, dendritic cells, and neutrophil cells in LGG (Fig. 8A), while no significant connections were found in GBM (Fig. 8B). In aspects of the tumor immune microenvironment, it presented significant associations with UNC5A in LGG (Fig. 8C) or GBM (Fig. 8D), according to our results. For immune checkpoints, the outcome of us discovered that UNC5A in GBM was positively correlated with ADORA2A, CD200, and negatively involved with CD44, CD48, while UNC5A in LGG was positively linked with CD200, TNFRSF18, TNFSF9, and negatively involved with BTLA, CD160, CD274, CD27, CD28, CD276, CD40LG, CD40, CD48, CD44, CD86, CD80, HAVCR2, CTLA4, LAIR1, ICOS, NRP1, LGALS9, PDCD1LG2, PDCD1, TNFRSF14, TMIGD2, TNFRSF9, TNFSF14, TNFSF4, VSIR (all p < 0.001; Fig. 8E). Regarding immune cells and UNC5A, the outcome of us discovered noteworthy correlations in LGG or GBM, containing the activated CD4 T cell route, the activated CD8 T cell route, and so on (all p < 0.001; Fig. 8F).

### UNC5A’s relationships with anticancer drug sensitivity

**Fig. 9:**
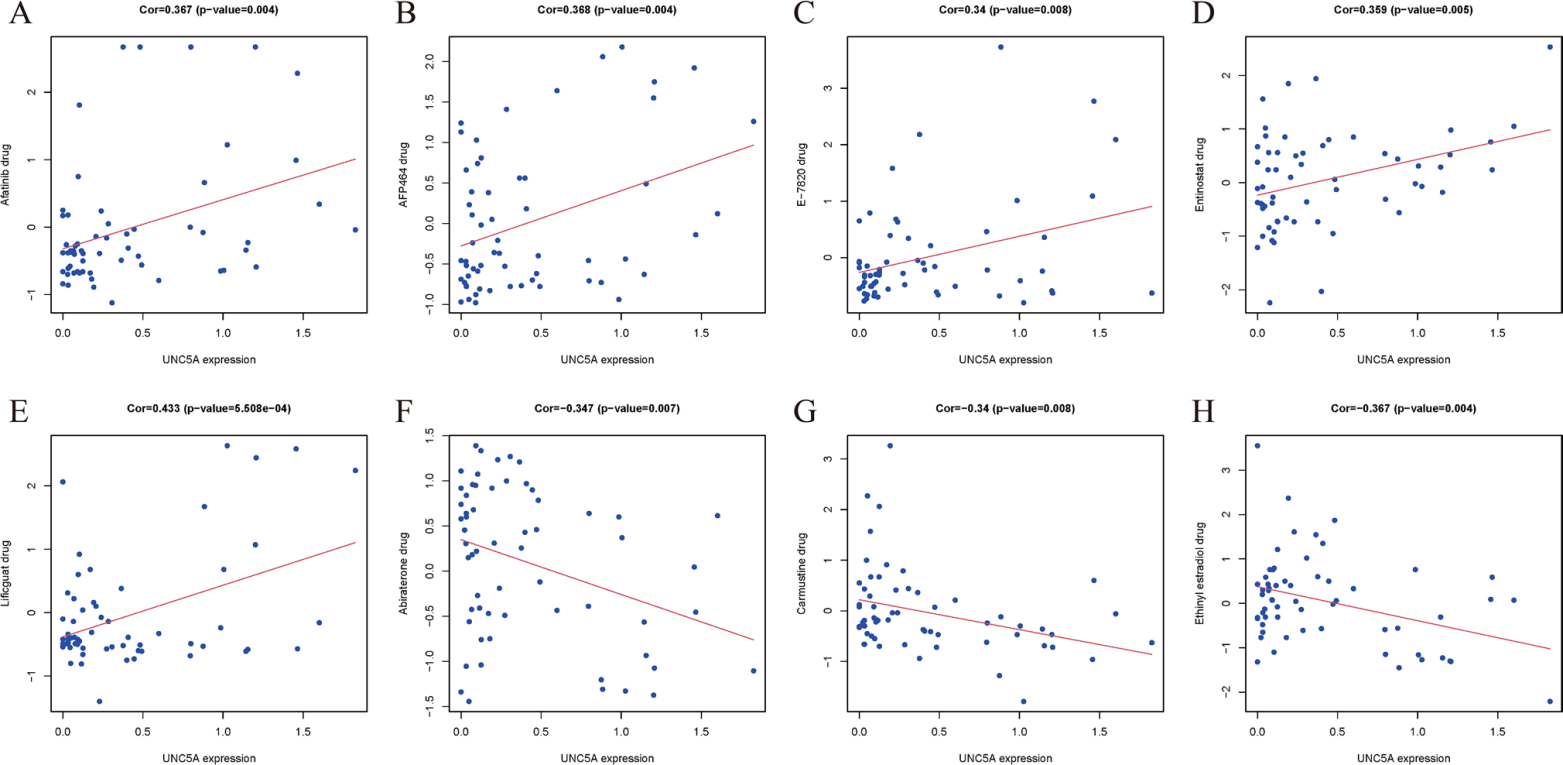
Relationships between UNC5A expression and anticancer drug sensitivity; **A** Afatinib; **B** AFP464; **C** E-7820; **D** Entinostat; **E** Lificguat; **F** Abiraterone; **G** Carmustine; **H** Ethinyl estradiol

The relationships among UNC5A and antitumor drug sensitivity was investigated via using the CellMiner database, with the threshold of |corFilter|≥ 0.3 and pFilter < 0.01. Finally, eight antitumor drugs significantly associated with UNC5A expression were selected out. Therein, UNC5A was positively correlated with Afatinib, Afp464, E-7820, Entinostat, Lificguat drug sensitivities, indicating patients with low UNC5A expression were more sensitive to these molecular drugs’ chemotherapies. UNC5A was negatively correlated with Abiraterone, Carmustine, Ethinyl estradiol drug sensitivities, indicating patients with high UNC5A expression were more sensitive to these molecular drugs’ chemotherapies (Fig. 9).

## Discussion

Significant advances in the screening, diagnosis, and treatment of glioma have been made in recent years, along with immunotherapy, stereotactic radiation, and novel chemotherapeutic agents being used in the clinic. However, its prognosis remained dismal [24]. UNC5A has been reported to play significant roles in the occurrence and progression of various tumors. Nevertheless, not much research has been done on UNC5A in gliomas up to this point. Therefore, in this study, we first combined scRNA-seq, proteomics, and bulk RNA-seq in order to investigate UNC5A's expression, prognosis, associated pathways or drugs, and clinical or immune relevance in gliomas.

According to our results, the TCGA dataset showed a significantly reduced expression of UNC5A by comparing gliomas with normal tissues, which was also confirmed by GSE50161, GSE4290, and QRT-PCR findings. In both the TCGA and CGGA datasets, gliomas patients with low-UNC5A expression would have poorer overall survival (OS) prognoses. ScRNA-seq analysis by the CancerSEA online website presented that UNC5A had a low expression in various glioma clusters and significantly associated with six functional states. Moreover, UNC5A might be a reliable independent biomarker of OS in gliomas patients. Based on the results of GSEA, UNC5A might be connected to three significant pathways in gliomas, including the calcium pathway, the Gnrh pathway, and the Jak-stat pathways. We also successfully created a Norman chart to assess the OS prognoses of these patients. Additionally, in aspects of tumor immunity, the infiltration levels of immune cells in LGG, the immune cell pathways, tumor immune microenvironment, as well as immune checkpoints in both LGG and GBM were revealed to be significantly influenced by UNC5A. Additionally, UNC5A was positively correlated with Afatinib, Afp464, E-7820, Entinostat, Lificguat drug sensitivities and negatively correlated with Abiraterone, Carmustine, Ethinyl estradiol drug sensitivities. All in all, UNC5A was found to have prognostic and immunological significance in gliomas, offering patients with gliomas new treatment options.

In order to acquire an understanding of how UNC5A contributes to the growth of gliomas, GSEA was conducted by us, and three signaling pathways involved with UNC5A in gliomas were identified: the calcium pathway, the GnRh pathway, and the Jak-stat pathway. The calcium signaling pathway was critical to cellular function and directly or indirectly involved in transmembrane cellular transport, thus affecting tumor invasion and metastasis [25]. The gonadotropin-releasing hormone (GnRH) signaling pathway controlled reproductive function as well as cancer growth and progression. GnRH regulated the release of various subordinate hormones and had a significant role in regulating the systemic microenvironment and endocrine regulation. It had been suggested that this pathway might interact with astrocyte [26]. Phosphorylation responses were mediated via the JAK/STAT signaling system, which had been linked to the pathophysiology of autoimmune and inflammatory diseases [27, 28]. Several cytokines that were implicated in the development of inflammatory and autoimmune disorders transduced intracellular signals through the usage of JAK and STAT. Research had demonstrated that neuroinflammatory illnesses such as multiple sclerosis and Parkinson's disease exhibited activation of the JAK/STAT pathway [24]. These all demonstrated the possible functions of the three UNC5A-related pathways in gliomas.

In order to better understand the functions that UNC5A played in tumor immunity, we mined the connections among UNC5A and immunological characteristics like TMB, MSI, TNB, tumor microenvironment, immune checkpoints, immune cell infiltrations, and immune cell pathways. According to our results, UNC5A presented significant associations with immune cell infiltrations in LGG and with immune checkpoints, tumor immune microenvironment, as well as immune cells in LGG or GBM. Upon analyzing immune checkpoints, we also discovered that LGG had significantly more significant immune checkpoints than GBM. GBM was shown to be only significantly associated with ADORA2A, CD200, CD44, and CD48. It had to be confirmed whether this suggested a more noteworthy clinical importance for UNC5A in LGG [29–33]. It had been shown that ADORA2A (adenosine A2a receptor) expression was associated with glioma development and it was found to be significantly more immunoreactive in gliomas containing infiltrating tumor cells than in normal brain tissue [32]. In their study of the effects of chondroitin sulfate on glioma, zhu et al. found that this drug promoted CD44 degradation, inhibited integrin β1 expression in glioma cells and thereby slowed down the aggressiveness of glioma, indicating a non-negligible role of CD44 in glioma [34]. Meanwhile, CD48 had also been studied and its high expression in gliomas might lead to a lower survival rate, while for CD200, its higher expression might prolong microglia activation and tumor growth. The same results were obtained in a Truncated form of CD200 (CD200S) study [35, 36]. The above evidence also indirectly indicated the immune mechanism that UNC5A might affect in glioma.

Finally, we investigated the relationships between UNC5A and oncologic drug sensitivity and identified eight drugs of interest. Therein, afatinib, a drug for non-small cell lung cancer, achieved its therapeutic goal through potent, irreversible dual inhibition of EGFR and HER2 tyrosine kinases [37, 38]. AFP464, inhibited angiogenesis by suppressing the expression of integrin alpha2 subunits in the endothelium, and also reported to have antitumor effects [39]. Entinostat had also demonstrated its antitumor effects through in vitro and animal studies [40]. Abiraterone, a mainstream drug in the treatment of prostate cancer, inhibited its development by inhibiting CYP17 and androgen production to achieve a chemical depot effect [41, 42]. Carmustine, was a common drug for the treatment of brain tumors and intracranial metastases, and Ethinyl estradiol was clinically valuable for the regulation of sex hormones [43]. These above three drugs had a negative correlation trend with UNC5A and they were commonly used in clinical practice. So, whether some new possibilities could be obtained through clinical studies to observe the chance of glioma development in users of the above drugs remained to be explored.

It was also important to acknowledge a few limitations. First, further in vivo and in vitro experiments were necessary to fully understand UNC5A’s activities in gliomas. Second, further clinical information was needed to investigate the relationships between UNC5A and immunological relevance, clinical relevance, and survival prognosis. Finally, in this article, UNC5A was measured by sequencing the glioma tumor biopsy. However, UNC5A measured by blood, urine, etc. had not been evaluated, and these shall be conducted in our subsequent articles. So, it was still too early to judge if UNC5A was a good biomarker for clinical applications.

## Conclusions

In conclusion, UNC5A was revealed to be a potential biomarker for glioma patients’ OS prognoses, potentially taking part in the progression of glioma via the calcium pathway, the Gnrh pathway, and the JAK-STAT pathway. Moreover, UNC5A exerted significant associations with immunity. All of these indicated the prognostic and immunological significance of UNC5A in gliomas, offering patients with gliomas new treatment options.

## Data Availability

RNA-sequencing data were downloaded from Chinese Glioma Genome Atlas (CGGA, https://www.cgga.org.cn) and verified in The Cancer Genome Atlas (TCGA, https://cancergenome.nih.gov/). All data supporting the findings of this study were available from the corresponding author upon reasonable request.
